# Carbon Mineralization of Sulfate Wastes Containing Pb: Synchrotron Pb M3-Edge XANES Analysis of Simultaneous Heavy Metal and Carbon Sequestration

**DOI:** 10.1021/acs.est.5c01640

**Published:** 2025-04-03

**Authors:** Jun Hu, Lauren N. Pincus, Dominik Wierzbicki, Yonghua Du, Catherine A. Peters

**Affiliations:** † Department of Civil and Environmental Engineering, Princeton University, Princeton, New Jersey 08544, United States; ‡ National Synchrotron Light Source II, Brookhaven National Lab, Upton, New York 11973, United States

**Keywords:** CCUS, trace element, solid waste, remediation, beneficiary use, vaterite, calcite

## Abstract

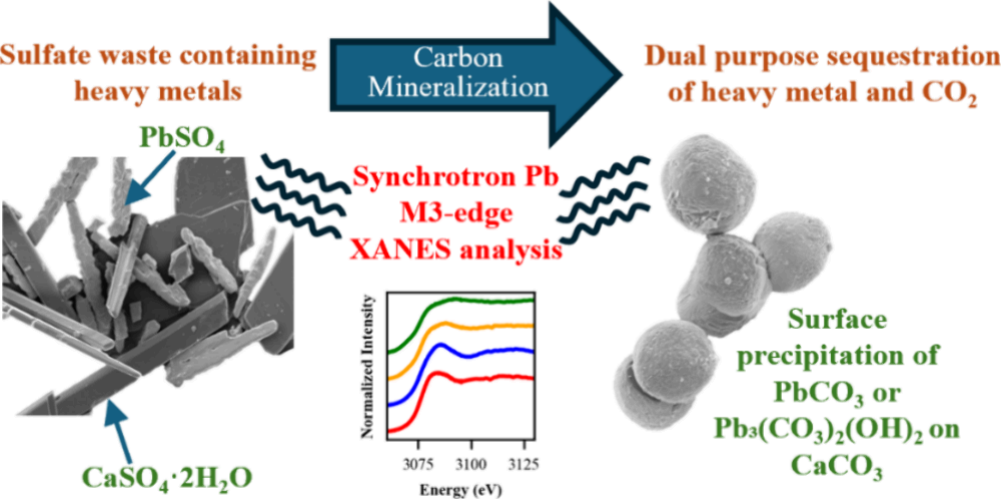

Sulfate wastes are produced in large quantities and contain toxic heavy metals such as lead (Pb), posing environmental risks. Because of favorable solubility differences, these wastes can be repurposed for engineered carbon dioxide (CO_2_) sequestration. Understanding the fate and mobility of heavy metals during this process is important. This study focuses on Pb and the effect of zinc (Zn) on Pb in carbon mineralization. Synthesized gypsum was treated with a carbonate-rich solution at pH 11.5 to convert the sulfates to carbonates. Aqueous solutions and mineral solids were analyzed. Synchrotron-based micro-X-ray fluorescence and a novel application of Pb M3-edge X-ray absorption near-edge structure provided detailed insights into Pb distribution and mineral forms. Results showed significant reductions in aqueous Pb and Zn concentrations, indicating effective metal sequestration. Carbon mineralization transformed Pb from soluble anglesite (PbSO_4_) into insoluble cerussite (PbCO_3_) and hydrocerussite (Pb_3_(CO_3_)_2_(OH)_2_). Pb primarily precipitated onto calcium carbonate surfaces through surface-mediated precipitation reactions. While the presence of Zn modified crystallization dynamics, it did not impede Pb sequestration and potentially enhanced surface reactivity, facilitating greater Pb immobilization. These findings highlight carbon mineralization as a sustainable approach to immobilize toxic metals in sulfate wastes while advancing CO_2_ sequestration efforts.

## Abstract 2

Carbon mineralization effectively transforms sulfate wastes containing toxic Pb into stable carbonate forms, thereby reducing metal toxicity and environmental hazards.

## 1 Introduction

Industrial and mining activities generate sulfate wastes, such as flue gas desulfurization gypsum (FGDG) and phosphogypsum. By 2020, global phosphogypsum production was estimated at 300 million tons per year, with projections indicating an increase to 438 million tons annually by 2050, driven by the growing demand for fertilizers to support global food production.^1^ FGDG production in the United States reached approximately 17 million tons in 2022,^2^ while in China, annual FGDG production exceeded 140 million tons as of 2023.^3^ These wastes, often enriched with toxic heavy metals, present considerable environmental and health risks.^4−8^ Despite the potential of being repurposed for beneficial uses, substantial quantities of sulfate wastes are still disposed of in landfills where inadequate containment often leads to the leaching of hazardous substances into soil and water systems.^2,9−11^ Given the abundance and hazards associated with sulfate wastes, effective management strategies are needed.

**Figure 1:**
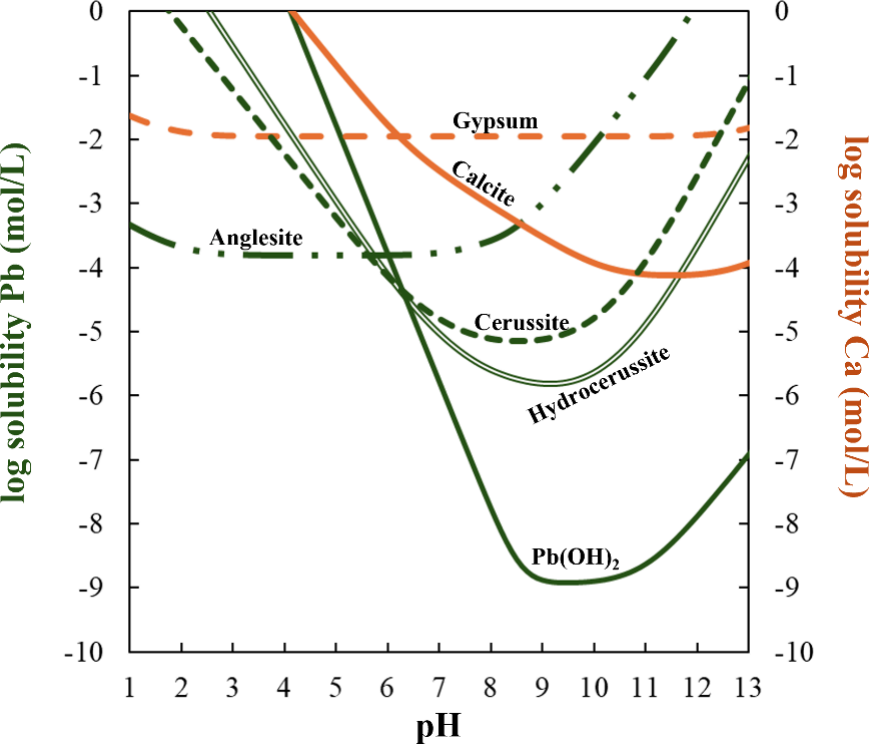
Aqueous solubility diagram of lead-bearing minerals (anglesite, cerussite, hydrocerussite, and Pb(OH)_2_) and calcium-bearing minerals (gypsum and calcite) for pH 1–13. Aqueous solubilities of individual minerals were calculated from PHREEQC (Version 3.7.3)^47^ simulations for a closed system at 25 °C. MINTEQ database^48^ was used.

Sulfate wastes, primarily composed of gypsum (CaSO_4_·2H_2_O), are well-suited for engineered carbon mineralization processes due to their high calcium content,^12−15^ offering a dual benefit of climate change mitigation and waste management. The carbon mineralization process involves the reaction of carbon dioxide (CO_2_) with materials rich in alkaline earth metals, resulting in the formation of stable carbonate minerals like calcite (CaCO_3_).^16−18^ The conversion of gypsum to calcite is thermodynamically favorable under near-neutral to basic pH conditions, driven by the lower solubility of calcite compared to gypsum (Figure 1). By applying carbon mineralization to the vast quantities of FGDG and phosphogypsum, it is estimated that up to 100 million tons of CO_2_ could be captured annually.^15^ This highlights the substantial potential of sulfate wastes to contribute to negative emission targets while advancing sustainable waste utilization and circular economy principles.

Despite the significant potential benefits of carbonation of sulfate wastes, the fate of toxic heavy metals like lead (Pb) remains insufficiently understood, posing a potential barrier to the widespread adoption of this strategy. Lead is a well-known neurotoxin that can cause developmental and neurological damage, emphasizing the need for robust strategies to mitigate Pb exposure.^19,20^ Anglesite (PbSO_4_) is frequently reported in sulfate-rich waste, where its low solubility raises environmental concerns regarding long-term stability and potential for Pb release.^21−23^ While previous studies have demonstrated that calcium carbonate coprecipitation can simultaneously sequester CO_2_ and heavy metals from highly contaminated solutions,^24−26^ it remains unclear whether this dual-purpose sequestration can be broadly achieved in solid waste systems.

In this work, we addressed the research questions to what extent engineered carbon mineralization can sequester the heavy metals in highly contaminated sulfate wastes and what mechanisms govern heavy metal sequestration during this process. Thermodynamic theory suggests that the dissolution of anglesite (PbSO_4_) at near-neutral to basic pH is expected to promote the formation of more stable lead carbonates, such as cerussite (PbCO_3_) or hydrocerussite (Pb_3_(CO_3_)_2_(OH)_2_) (Figure 1).^27,28^ While Pb(OH)_2_ is predicted to be the most stable phase under highly alkaline conditions, kinetic limitations may hinder its precipitation.^29^ The formation of relatively insoluble Pb carbonate minerals suggests that carbon mineralization can effectively immobilize Pb by precipitating it as carbonate phases under alkaline conditions.^28,30−32^ Carbonation of PbSO_4_ has previously been shown to achieve conversions greater than 90%, highlighting its potential as a viable strategy for stabilizing Pb-bearing sulfate wastes.^33^ Previous studies have also shown that calcite can incorporate certain divalent metal cations through isomorphic substitution, thereby enhancing metal uptake from the aqueous phase.^24,25,34−36^ However, the formation of solid solutions between Pb and Ca carbonates is limited by their different crystal structures and the larger ionic radius of Pb relative to Ca,^37^ making the incorporation of Pb into the calcite lattice unfavorable. Recent research has provided insights into the interactions between Pb and calcite surfaces, suggesting that surface incorporation or mineral replacement processes can facilitate Pb immobilization despite structural differences.^30,31,38,39^ This study seeks to explore these processes to better understand Pb dissolution and precipitation in an environmental remediation context, focusing on the potential for Pb mobilization from sulfate minerals and its transformation during carbon mineralization.

We investigated the fate of Pb during the carbonation of sulfate precipitates. Synthesized Pb-containing gypsum was used as a model system to simulate sulfate wastes, allowing investigation of the mechanisms of Pb immobilization. The effectiveness of heavy metal sequestration was evaluated by measuring the residual concentrations of heavy metals in the aqueous phase. Morphological transformations were studied using scanning electron microscopy (SEM), while the spatial distribution of metals was analyzed using synchrotron-based micro-X-ray fluorescence (μXRF).

X-ray absorption near-edge structure (XANES) spectroscopy is a powerful technique for analyzing the electronic structure, oxidation state, and local coordination of elements in diverse materials. For Pb, the L3-edge XANES is well-established and widely applied in various fields.^40−43^ However, the Pb L3-edge (13.04 keV) requires hard X-ray beamlines, which often cannot analyze lighter elements such as Ca (K-edge 4.04 keV) and S (K-edge 2.47 keV) within the same experimental setup. The Pb M-edge XANES, situated in the tender-energy range, provides an alternative for multielement studies. The Pb M5-edge (2.48 keV) has been explored due to its strong spectral features and sensitivity to core electron structure.^44−46^ However, a significant limitation of the Pb M5-edge is its fluorescence overlap with the S K-edge. This overlap of signal complicates analyses in sulfur-rich matrices, such as sulfates or sulfur-containing environmental samples.

A key contribution of this study is the advancement of XANES for Pb mineral analysis. We utilized the TES beamline at the National Synchrotron Light Source II (NSLS II), which operates in the 2–5.5 keV energy range spanning the sulfur (S) K-edge, calcium (Ca) K-edge, and lead (Pb) M-edge. Excitation energies for these elements are summarized in Table S1 (see Supporting Information). This tender X-ray beamline is uniquely suited for simultaneous measurements of S and Pb. To overcome the challenges of measuring Pb in the tender X-ray energy range, we employed a novel approach using Pb M3-edge XANES, enabling the identification of Pb mineral phases in complex matrices, including those rich in S. This method was applied to study Pb mineral phases in sulfate waste before and after carbon mineralization, providing valuable insights into the transformation of Pb minerals and the sequestration of Pb into lower-solubility phases.

To account for the complexity of sulfate wastes, this study also investigated the influence of foreign cations, such as zinc (Zn), on Pb behavior during carbonation. Zinc is often present in industrial and mining waste streams at concentrations that can be toxic to aquatic and terrestrial organisms, making its mobility and eventual fate in the environment a concern.^5,22^ Foreign cations like Zn may compete with Pb for carbonate ions, potentially affecting Pb immobilization by either promoting its incorporation into carbonate minerals or enhancing its mobility. Further investigation into the role of competing cations is needed to predict the effectiveness of mineral carbonation in immobilizing Pb in sulfate wastes that often contain a mixture of metal cations.

## 2 Materials and Methods

### 2.1 Experimental Setup

Calcium sulfate precipitates were synthesized to represent Pb-containing gypsum. A solution containing 62.5 mM CaCl_2_·2H_2_O, 62.5 mM Na_2_SO_4_, and 5 mM Pb(NO_3_)_2_ was the initial condition in the sulfate precipitation experiments. The Pb concentration was intentionally set higher than environmental levels to ensure supersaturation with respect to lead sulfate, thereby facilitating sufficient Pb precipitate formation in the solid phase for characterization and analysis. The initial pH of the solution was measured at pH ≈ 5.6 after equilibration. A parallel set of experiments included Zn, with a final concentration of 5 mM ZnCl_2_. For selected experiments, following sulfate precipitation, a carbonate-rich solution was added, and the molar ratio of Na_2_CO_3_ to the initially introduced Ca was 1:1. All experiments were conducted in sealed vessels with minimal headspace to minimize CO_2_ volatilization. The pH was adjusted to 11.5 by addition of NaOH to promote calcite precipitation. All chemicals used were ACS-grade to ensure consistency and purity across experiments.

The reaction mixtures were initially agitated at 23 °C on a shaker table for 2–3 h, followed by quiescent equilibration for the remainder of the seven-day reaction period. The reaction period was chosen based on a kinetic study of gypsum dissolution coupled with CaCO_3_ precipitation at room temeprature.^49^ Aqueous solution samples were filtered through a 0.2 μm glass fiber filter and acidified with 2% nitric acid to stabilize metal ions. Aqueous samples were analyzed using inductively coupled plasma mass spectrometry (ICP-MS) on a Thermo Neptune multicollector, achieving detection limits in the ng/L range. Double replicate experiments were performed, and average concentrations are reported.

### 2.2 Solid Phase Characterization

The solid precipitates collected from each batch were rinsed three times with deionized water and ethanol, then dried for analysis. Morphological examination was conducted using SEM at 5 keV under high vacuum with a Quanta 200 FEG Environmental SEM. Backscattered electron (BSE) imaging was employed for compositional contrast, particularly effective for detecting heavier elements, while secondary electron (SE) imaging provided detailed surface topography and morphological information. The bulk XRD analysis was not performed, as the metal concentrations in the solid phase were at trace levels and below the detection limit of the technique.

Solid precipitates were analyzed at the NSLS-II tender energy X-ray beamline (TES, 8-BM) for μ-XRF and Pb M3-edge XANES spectroscopy. Because Zn K-edge and L-edges fall outside the TES beamline’s operational energy range (Table S1), Zn could not be detected in the solids at this beamline. An X-ray beam with a spot size of 0.5 mm × 0.5 mm was used using a set of K–B mirrors. The incident beam energy was selected using a Si (111) double crystal monochromator. Small amounts of solid precipitate powders were spread on sulfur-free Kapton tape for measurements. XANES spectra were recorded in fluorescence mode using a silicon drift detector (Canberra).

μXRF maps were collected at an excitation energy of 4.4 keV over a 300 μm × 300 μm scan area, with a dwell time of 0.1 s per point and a step size of 2 μm. Data were processed using PyXRF software,^50^ and image postprocessing was performed using FIJI.^100^ Elemental correlations within the samples were visualized using XMIDAS,^51^ which allowed us to identify how different elements are spatially associated with one another.

For XANES analysis, Pb M3-edge scans were collected from specific locations of interest that were identified via μXRF. The scan range for the Pb M3-edge scans was 2980–3190 eV, with a step size of 2 eV between 2980 and 3040 eV, 1 eV between 3040 and 3060 eV, 0.3 eV between 3060 and 3100 eV, and 1 eV between 3100 and 3190 eV, with a dwell time of 3 s per point. At least three scans were collected for each sample to improve accuracy and precision. Beam damage was carefully monitored and not detected during these repeat scans. Reference Pb mineral standards included anglesite (PbSO_4_), cerussite (PbCO_3_), hydrocerussite (Pb_3_(CO_3_)_2_(OH)_2_), and Pb(OH)_2_. Mineral standards were analyzed at the SMART XAS endstation of the TES beamline. The standards were prepared in the form of pellets diluted to 5% Pb by weight using polyethylene glycol to avoid self-absorption. At least 15 scans were collected and averaged for each standard. The collected XANES scans were merged, background-subtracted and normalized using the Athena software package.^52^ Due to the trace amounts of Pb in the measured samples, the point scans of specific spots exhibited some noise. A Gaussian filter algorithm was applied to these scans to reduce noise. Figure S1 in the Supporting Information illustrates an example of raw and smoothed data.

## 3 Results and Discussion

### 3.1 Aqueous Phase Analyses

**Figure 2:**
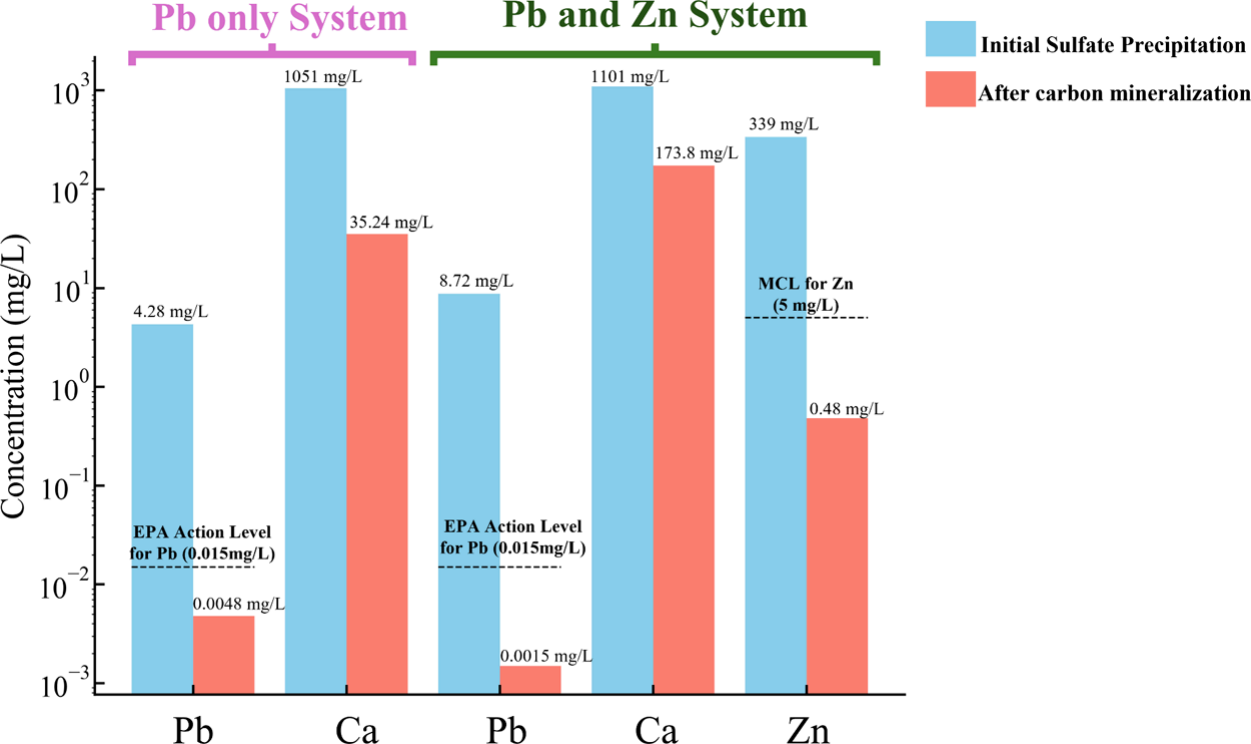
Residual aqueous concentrations of Pb and Zn after sulfate precipitation (blue) and after carbon mineralization (red) for solutions containing only Pb and solutions containing both Pb and Zn. Dashed lines indicate the U.S. EPA action level for Pb (0.015 mg/L) and the maximum contaminant level (MCL) for Zn (5 mg/L). The *y*-axis is plotted on a logarithmic scale. Measurements were performed in duplicate, but the error bars are too small to be visible on the plot.

The effectiveness of heavy metal sequestration through carbon mineralization was evaluated by measuring the residual concentrations of Pb and Zn in the aqueous phase after sulfate precipitation and subsequent carbon mineralization (Figure 2). The sulfate precipitates, containing either Pb alone or both Pb and Zn, demonstrated limited ability to immobilize Pb due to the relatively high solubility of sulfate minerals. This resulted in high residual metal concentrations in the aqueous phase after precipitation. In the system containing Pb only, the residual aqueous Pb concentration was 4.28 mg/L, whereas in the system containing both Pb and Zn, the residual concentrations were 8.72 mg/L for Pb and 339 mg/L for Zn. These concentrations greatly exceed the U.S. EPA action level for Pb (0.015 mg/L) and the maximum contaminant level (MCL) for Zn (5 mg/L).^53,54^

Following carbon mineralization, the aqueous concentrations of Pb and Zn decreased significantly (Figure 2). The final Pb concentration in the system containing Pb only was 0.0048 mg/L, whereas in the system containing both Pb and Zn, Pb decreased to 0.0015 mg/L and Zn to 0.48 mg/L. These reductions highlight the efficiency of carbon mineralization in immobilizing heavy metals, bringing residual concentrations well below regulatory limits. The final Pb concentrations were lower than the U.S. EPA action level for Pb (0.015 mg/L), while Zn concentrations also fell below its MCL (5 mg/L). The marked decrease in metal concentrations after carbon mineralization reflects the formation of low-solubility carbonate minerals under alkaline conditions, effectively sequestering Pb and Zn from the aqueous phase.

Interestingly, the presence of Zn appears to enhance Pb sequestration during carbon mineralization, as indicated by the lower residual Pb concentration compared to the Pb-only system. Analysis of the residual Ca concentrations provides insight into the potential mechanisms underlying this enhancement (Figure 2). In the Pb-only system, the residual Ca concentration was 35.24 mg/L, whereas in the system containing Zn, it was significantly higher (173.8 mg/L). This difference could result from Zn substituting for Ca in the carbonate precipitates, leaving more Ca in solution. Previous studies have demonstrated that Zn can form a solid solution with calcite through coprecipitation.^24,55,56^ Solid solution–aqueous solution (SS–AS) theory indicates that Zn incorporation reduces the mole fraction of calcite within the solid phase, consequently increasing the equilibrium concentration of Ca in the aqueous phase.^36^ The substitution of Zn into the carbonate matrix could alter the crystal properties, potentially stabilizing defects, increasing surface roughness, or modifying the lattice structure.^55,57^ These changes may create additional sites that promote Pb coprecipitation.

### 3.2 Lead Mineral Standard for M3-Edge XANES

The Pb M3-edge XANES spectra recorded for the mineral standards are shown in Figure S2. These standards exhibited distinct and reproducible spectral features that enable precise differentiation between mineral standards. The absorption edges are observed at 3084.87 eV (anglesite) < 3085.77 eV (cerussite) < 3087.58 eV (hydrocerussite) < 3092.09 eV (lead hydroxide). Anglesite and cerussite feature sharp peaks and a steep postedge decline, whereas hydrocerussite and lead hydroxide exhibit broader peaks and a gradual postedge decline. These differences in spectral characteristics of these standards provide clear features for identification of each Pb phase.

### 3.3 Solid Phase and Spatial Analyses

SEM-SE imaging (Figure S3) revealed pronounced morphological transformations in the precipitates in response to carbon mineralization and the presence of Zn. The Pb-containing sulfate precipitates displayed needle-like crystals, a morphology characteristic of gypsum, which typically forms elongated, prismatic structures due to its monoclinic crystal system and layered hydration.^58−60^ The morphology of the Pb-containing sulfate precipitates aligns with this characteristic gypsum structure, which often appears as acicular or tabular crystals. After carbonation, the precipitated solids are cubic-like particles with rhombohedral facets, indicative of calcite formation, as calcite commonly manifests in this distinctive rhombohedral morphology due to its trigonal crystal system.^61^ This morphological shift signifies the conversion of calcium sulfate to calcium carbonate, aligning with thermodynamic predictions and previous observations in similar systems.^13−15^

In the sample containing both Pb and Zn, the SEM images showed the formation of larger and nearly spherical particles after carbonation. These sphere-like shapes differ from the rhombohedral calcite observed in the Pb-only system and suggest an alteration in the crystal structure due to the presence of Zn. In addition to forming a solid solution with calcite, Zn can also influence the crystallization pathway of CaCO_3_. The effects of Zn on the crystallization of CaCO_3_ have been observed in other studies.^24,55,62−64^ This could occur through adsorption of Zn ions on growth sites, which can limit crystal growth rates and alter the resulting morphology.^24,64,65^ The vaterite-like structure observed here is consistent with previous findings where Zn promotes metastable phases, potentially delaying transformation to more stable calcite structures.^63,64,66^ This alteration in growth and stability dynamics suggests a potential enhancement in the persistence of metastable phases.

**Figure 3:**
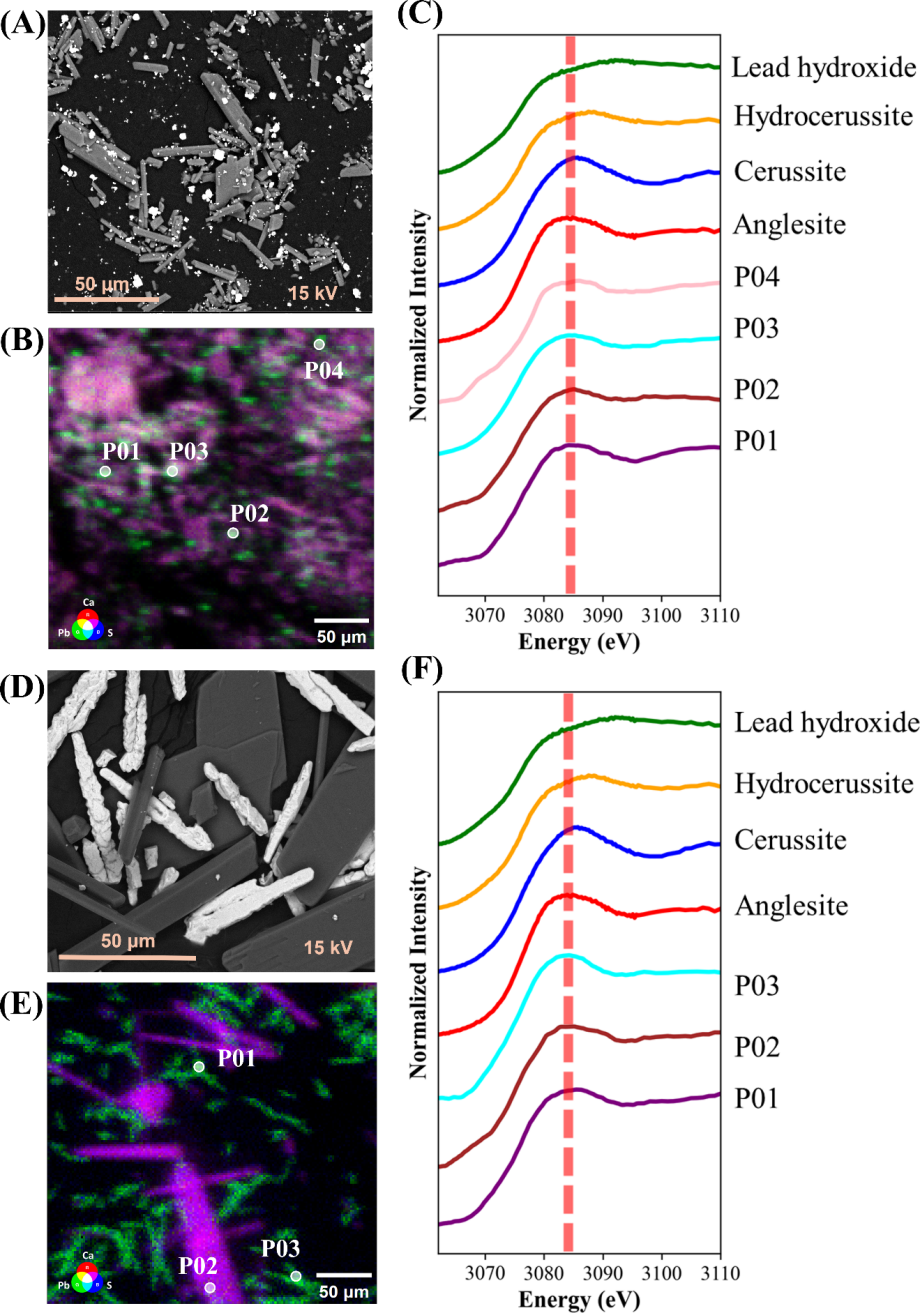
SEM-BSE images of gypsum precipitates containing (A) only Pb and (D) both Pb and Zn. μXRF elemental maps of Ca, S, and Pb for gypsum precipitates containing (B) only Pb and (E) both Pb and Zn. Normalized Pb M3-edge XANES scan from selected points in gypsum precipitates containing (C) only Pb and (F) both Pb and Zn. The red dashed line indicates the absorption edge position of the anglesite standard.

SEM-BSE imaging and three-element μXRF elemental mapping were used to elucidate the spatial distribution of Pb within the precipitates. In BSE images, heavier elements appear brighter due to increased backscatter intensity. In the sulfate precipitates containing Pb only, the BSE image shows rhombus-shaped crystals that appeared brighter than the gypsum crystals (Figure 3A), indicating Pb enrichment. Similar morphological patterns were reported by Astilleros et al.^27^ on the interaction of gypsum with lead in aqueous solutions, suggesting that these particles are anglesite. The μXRF mapping further confirmed that Pb was present as isolated particles, distinct from Ca signals associated with gypsum (Figure 3B). This suggests that Pb did not incorporate into the gypsum lattice but precipitated as a separate phase. Pb M3-edge XANES scans of high-Pb locations (P01–P04) contain spectral features that closely resemble those of the anglesite standard (Figure 3C), with a peak at ∼3085 eV, confirming that Pb primarily existed as a sulfate mineral in the initial experimental stage.

In sulfate precipitates containing both Pb and Zn, BSE images revealed discrete bright particles with rough, elongated, and pointed structures (Figure 3D). μXRF mapping indicated that these bright particles contained high Pb and S signals, confirming Pb remained as isolated particles without significant spatial overlap with Ca (Figure 3E). The XANES scans continued to match the anglesite standard (Figure 3F), confirming that the presence of Zn did not alter the mineral form of Pb. The morphological shift may be attributed to changes in the solution environment introduced by Zn, potentially influencing the growth dynamics of Pb sulfate crystals. Similar morphologies have been noted in other studies, for instance, Yang et al.^67^ observed thorn-like and dendritic PbSO_4_ crystals with high aspect ratios formed under controlled conditions, demonstrating how factors like concentration and ligand presence can significantly modify Pb sulfate morphology.

**Figure 4:**
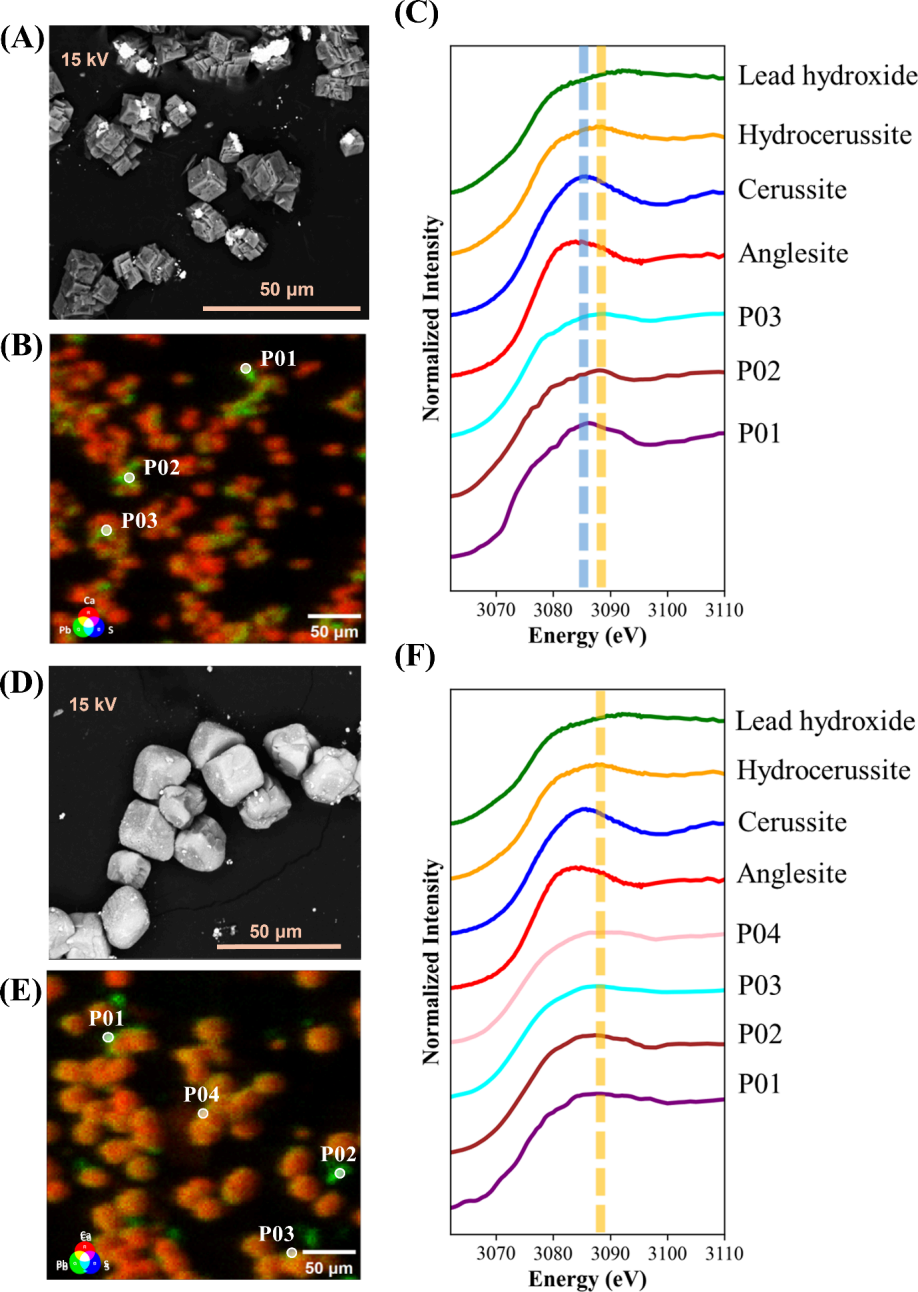
SEM-BSE images of carbonated precipitates containing (A) only Pb and (D) both Pb and Zn. μXRF elemental maps of Ca, S, and Pb for carbonated precipitates containing (B) only Pb and (E) both Pb and Zn. Normalized Pb M3-edge XANES scan from selected points in carbonated precipitates containing (C) only Pb and (F) both Pb and Zn. The yellow dashed line indicates the absorption edge position of the hydrocerussite standard, while the blue dashed line indicates the absorption edge position of the cerussite standard.

In the carbonated solid precipitated from solution containing only Pb, BSE images showed that bright particles remained discrete from the calcite particles (Figure 4A). These bright particles varied in size and exhibited irregular shapes, scattered on or adjacent to the rhombohedral calcite particles. μXRF mapping confirmed that Pb was distributed as isolated particles (Figure 4B). Three points with high Pb fluorescence counts were selected for analysis, and Pb M3-edge XANES scan revealed Pb in carbonate mineral form. The scan from point P01 shared spectral features with the cerussite standard, with a peak at ∼3086 eV, while the scans from points P02 and P03 exhibited spectral features consistent with hydrocerussite (Figure 4C). Figures S4 and S5 (Supporting Information) are the absorption-edge position analyses of the points in Figures 3 and 4, respectively. In Figure S4, the points are positioned near anglesite, while in Figure S5, all measured points shift toward positions closest to cerussite or hydrocerussite. This shift in absorption edge position suggests that carbonation facilitated the transformation of Pb from sulfate to less soluble carbonate phases. The formation of cerussite and hydrocerussite aligns with the expected precipitation of Pb carbonates under alkaline conditions with high carbonate availability. The fact that Pb-rich particles remained discrete from calcite particles suggests limited bulk incorporation of Pb into the calcite lattice due to differences in ionic radius and crystal structure between Pb and Ca.^68^

In the carbonated solid precipitated from a solution containing both Pb and Zn, BSE images showed slightly brighter (light gray) CaCO_3_ particles compared to those in the Pb-only case, likely due to the coprecipitation of Zn within the CaCO_3_ lattice as a solid solution, resulting in a higher atomic mass and increased backscatter intensity (Figure 4D). These findings are consistent with prior studies that demonstrate Zn incorporation into calcite through coprecipitation processes, facilitating its uptake in waste treatment applications.^24,57,65^ In addition, smaller and brighter (white) spherical particles were observed around the larger, near-spherical CaCO_3_ particles. The larger size of Zn-modified CaCO_3_ particles improved visualization of elemental distribution and colocalization in μXRF maps, revealing that Pb was predominantly colocated with Ca, with some regions indicating discrete Pb precipitation. This colocation of Pb and Ca within Zn-modified CaCO_3_ is intriguing, as it contrasts with existing theories suggesting that Pb is unlikely to form solid solution within the CaCO_3_ lattice due to differences in ionic radius and crystal structure between Pb and Ca. The observed colocation raises questions about potential mechanisms by which Pb may interact with CaCO_3_ under specific conditions. Four points exhibiting high Pb fluorescence signal were selected for XANES analysis: P01, P03, and P04 were located on the CaCO_3_ particles, while P02 was an isolated particle. The XANES scans at each point contain spectral features that appear to match those of the hydrocerussite standard (Figure 4F), with a distinct peak at ∼3088 eV followed by a gradual decay, indicating that Pb in these precipitates predominantly existed as a carbonate phase. The absorption-edge positions of measured points in Figure 4F were also plotted in Figure S5, showing they are positioned closest to hydrocerussite. This formation of low-solubility Pb carbonates suggests that Zn did not interfere with the Pb transformation process during carbonation, allowing Pb to stabilize in less soluble carbonate phases. The presence of Pb as hydrocerussite implies a reduction in Pb mobility and an enhancement in environmental stability within the carbonated matrix.

### 3.4 Colocation of Pb and Ca

**Figure 5:**
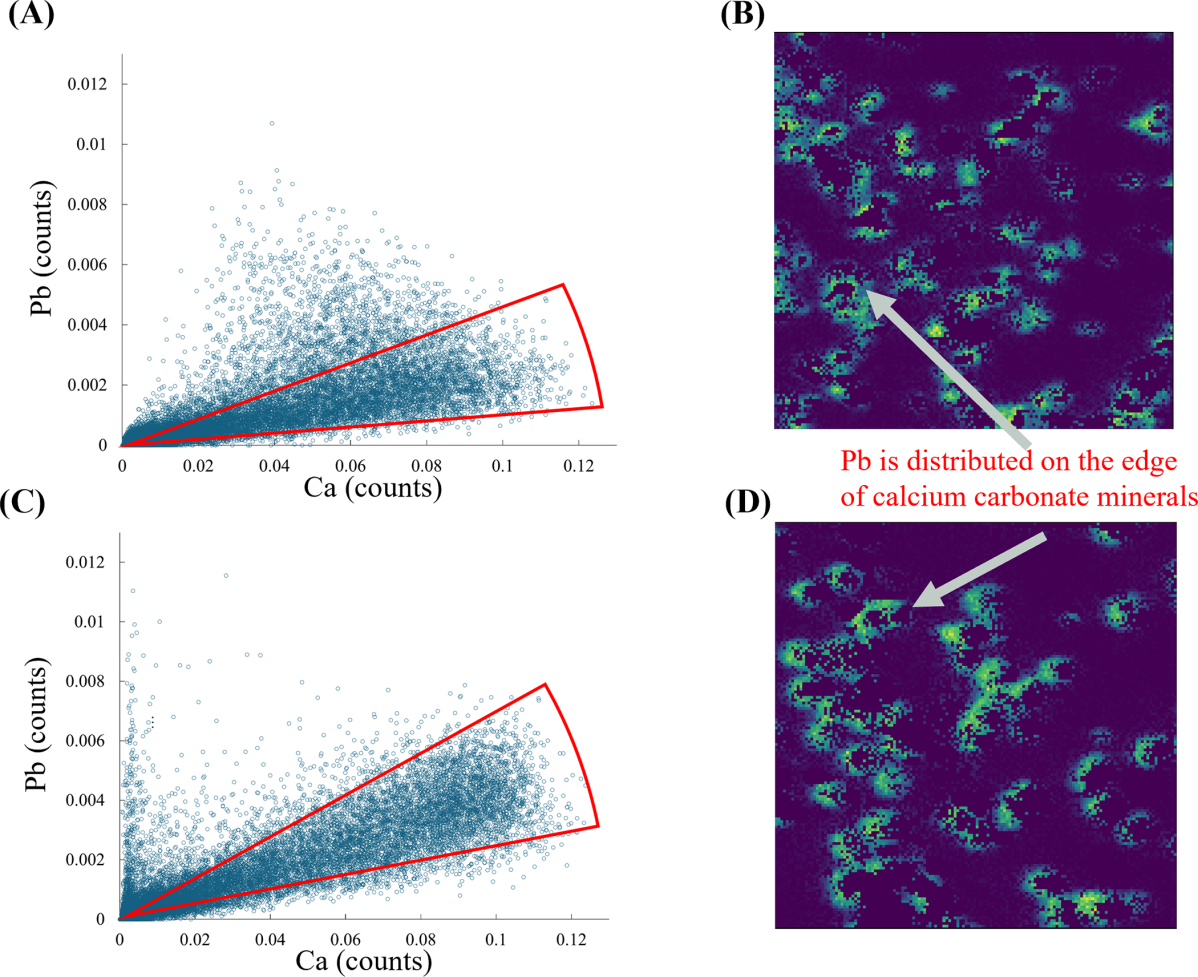
Element correlation plots for Ca and Pb in carbonated precipitates containing (A) only Pb and (C) both Pb and Zn. Regions of interest with the strongest correlation are highlighted within triangular-shaped areas. Corresponding single-element μXRF for Pb in carbonated precipitates containing (B) only Pb and (D) both Pb and Zn.

Element correlation analyses were conducted to explore the colocation of Pb and Ca in the carbonated precipitates. Figure 5A presents the correlation plot of normalized μXRF fluorescence counts in pixels across the entire scan area for Ca and Pb, while Figure 5C shows the corresponding plot for samples containing Zn. The highest density data are highlighted within triangular-shaped regions, showing the compositions that are most strongly correlated. The Zn slightly increased the ratio of Pb to Ca, a result that is consistent with the aqueous phases analyses which showed lower aqueous Pb concentration when Zn was present.

The corresponding single-element μXRF maps are shown in Figure 5B,D, where increasing yellow coloration indicates higher Pb fluorescence counts. The strong positive correlation between Ca and Pb fluorescence counts, as seen in the highlighted regions, suggests colocalization of Pb and Ca within the precipitates. This correlation might be interpreted as coprecipitation of Pb with Ca. However, examination of the Pb μXRF maps (Figure 5B,D) reveal that Pb is predominantly distributed on the surfaces and edges of the CaCO_3_ crystals rather than uniformly incorporated throughout the calcite. This spatial distribution indicates that Pb does not form a solid solution with calcite but is instead localized at or near the surfaces of calcite crystals.

The observation that Pb deposits on the surfaces of CaCO_3_ crystals suggests that Pb immobilization occurs primarily through surface-mediated precipitation of Pb carbonate phases, such as cerussite or hydrocerussite, on CaCO_3_ mineral surfaces. The formation of these Pb carbonate minerals effectively sequesters Pb, reducing its mobility in the environment. Our observations corroborate earlier studies employing advanced imaging techniques, such as synchrotron transmission X-ray microscopy (TXM). Yuan et al.^31^ used TXM to reveal that Pb replaces Ca in calcite through a dissolution–reprecipitation process, forming a porous cerussite shell around the calcite core in a pseudomorphic manner—maintaining the external morphology of the original calcite crystal. Similarly, Kim et al.^30^ employed TXM to observe the formation of cerussite layers on the surfaces of calcite crystals under acidic conditions, demonstrating a pseudomorphic replacement process. Furthermore, Abdilla et al.^38^ demonstrated that Pb can dynamically incorporate into dissolving calcite surfaces under far-from-equilibrium conditions, forming a Pb-rich surface layer without full lattice integration. The presence of Zn did not significantly affect the localization of Pb on the calcite crystal, as indicated by similar correlation patterns and XANES scan in both the Pb-only and Pb–Zn samples (Figure 5C,D).

## 4 Environmental Implications

This study demonstrates the practical potential of engineered carbon mineralization to address two pressing environmental challenges: managing hazardous sulfate wastes and mitigating CO_2_. Sulfate wastes pose significant environmental risks to soil and water systems due to heavy metal leaching. The carbon mineralization process investigated here transforms these wastes into stable carbonate minerals, while simultaneously immobilizing heavy metals like Pb and reducing their environmental mobility.

This research demonstrates that Pb immobilization during carbon mineralization is achieved through its transformation into low-solubility carbonate phases, such as cerussite and hydrocerussite. The crystal structural differences between lead and calcium carbonates make the formation of solid solutions between lead and calcium carbonate unfavorable. This research showed that surface precipitation is the key pathway in which Pb preferentially localizes on calcium carbonate particles, where surface-mediated precipitation reactions drive the formation of a Pb-rich carbonate layer. This mechanism stabilizes Pb in mineral forms with low solubility, thereby preventing its release into the environment. In addition, the presence of coexisting cations, such as Zn, modifies crystallization dynamics but does not impede Pb sequestration. Instead, Zn potentially enhances surface reactivity and facilitates further Pb immobilization.

A comprehensive economic evaluation and life cycle assessment specific to sulfate waste carbon mineralization, including detailed assessments of reagent consumption, energy use, infrastructure, and scalability, will be necessary for practical implementation. However, carbon mineralization processes show promising economic feasibility, particularly when CO_2_ can be sourced directly from industrial flue gases, significantly reducing reagent costs and overall energy demand for CO_2_ purification.^69−72^ Compared with other metal stabilization techniques such as cement stabilization and biochar amendments, carbon mineralization offers additional benefits like permanent CO_2_ sequestration and potential reuse of treated wastes, making it a competitive option for heavy metal stabilization.

The innovative application of Pb M3-edge XANES in this study enables characterization of Pb mineral form during carbon mineralization. This technique effectively overcomes analytical challenges associated with fluorescence overlap of other commonly used Pb edges within the tender region, such as the M5-edge, with sulfur K-edge, allowing for detailed insights into Pb phase transformations. Specifically, Pb M3-edge XANES can distinguish between various Pb phases, providing a deeper understanding of the mechanisms driving Pb sequestration in carbon mineralization. Beyond this study, Pb M3-edge XANES holds significant potential for application in other fields, including geochemistry, material science, and environmental remediation, where precise characterization of Pb phases that co-occur with S is required.

## Supporting Information Available

X-ray absorption energy of Ca, S, Pb, and Zn; example comparing raw and smoothed Pb M3-edge XANES data shown for point P03 in carbonated precipitates containing both Pb and Zn; Pb M3-edge XANES spectra of lead reference materials; SEM-SE images of initial gypsum precipitates and carbonated precipitates; absorption-edge positions (energy vs normalized μ(E)) of selected points from Figure 3C,F, compared with reference standards; absorption-edge positions (energy vs normalized μ(E)) of selected points from Figure 4C,F, compared with reference standards; overlay comparison of XANES spectra for selected points (P01–P04) in Figure 4F with the hydrocerussite reference spectrum; element correlation plots of normalized μ-XRF counts in initial sulfate precipitates containing only Pb; and element correlation plots of normalized μ-XRF counts in initial sulfate precipitates containing both Pb and Zn (PDF)

## Abbreviation

XANES: X-ray absorption near edge structure
SEM: scanning electron microscope
ICP-MS: inductively coupled plasma mass spectrometry
μXRF: micro X-ray fluorescence

## References

[ref1] Nedelciu C. E.; Ragnarsdottir K. V.; Schlyter P.; Stjernquist I. Global Phosphorus Supply Chain Dynamics: Assessing Regional Impact to 2050. Glob. Food Secur. 2020, 26, 100426 10.1016/j.gfs.2020.100426. PMC7490587 32953430

[ref2] 2022 Coal Combustion Product (CCP) Production & Use Survey Report, 2023.

[ref3] Wang H.; Wang F.; Qin W.; He C.; Wang F.; Liang X.; Li X. A Critical Review on the Use of Flue Gas Desulfurization Gypsum to Ameliorate Saline-Alkali Soils and Its Prospect for Reducing Carbon Emissions. Sci. Total Environ. 2024, 945, 174053 10.1016/j.scitotenv.2024.174053. 38897464

[ref4] Tayibi H.; Choura M.; López F. A.; Alguacil F. J.; López-Delgado A. Environmental Impact and Management of Phosphogypsum. J. Environ. Manage. 2009, 90 (8), 2377–2386. 10.1016/j.jenvman.2009.03.007. 19406560

[ref5] Hao Y.; Li Q.; Pan Y.; Liu Z.; Wu S.; Xu Y.; Qian G. Heavy Metals Distribution Characteristics of FGD Gypsum Samples from Shanxi Province 12 Coal-Fired Power Plants and Its Potential Environmental Impacts. Fuel 2017, 209, 238–245. 10.1016/j.fuel.2017.07.094.

[ref6] Wenyi T.; Suping G.; Wei X.; Youxu L.; Zixin Z. Feature Changes of Mercury during the Carbonation of FGD Gypsum from Different Sources. Fuel 2018, 212, 19–26. 10.1016/j.fuel.2017.09.119.

[ref7] Rutherford P. M.; Dudas M. J.; Samek R. A. Environmental Impacts of Phosphogypsum. Sci. Total Environ. 1994, 149 (1), 1–38. 10.1016/0048-9697(94)90002-7.

[ref8] Rutherford P. M.; Dudas M. J.; Arocena J. M. Heterogeneous Distribution of Radionuclides, Barium and Strontium in Phosphogypsum by-Product. Sci. Total Environ. 1996, 180 (3), 201–209. 10.1016/0048-9697(95)04939-8.

[ref9] Pérez-López R.; Macías F.; Cánovas C. R.; Sarmiento A. M.; Pérez-Moreno S. M. Pollutant Flows from a Phosphogypsum Disposal Area to an Estuarine Environment: An Insight from Geochemical Signatures. Sci. Total Environ. 2016, 553, 42–51. 10.1016/j.scitotenv.2016.02.070. 26901801

[ref10] Du X.; Li X.; Feng Q.; Meng L.; Sun Y. Environmental Risk Assessment of Industrial Byproduct Gypsum Utilized for Filling Abandoned Mines. Int. J. Coal Sci. Technol. 2022, 9 (1), 56 10.1007/s40789-022-00520-1.

[ref11] Shi X.; Zeng A.; Duan H.; Zhang H.; Yang J. Status and Development Trends of Phosphogypsum Utilization in China. Circ. Econ. 2024, 3 (4), 100116 10.1016/j.cec.2024.100116.

[ref12] Akfas F.; Elghali A.; Aboulaich A.; Munoz M.; Benzaazoua M.; Bodinier J.-L. Exploring the Potential Reuse of Phosphogypsum: A Waste or a Resource?. Sci. Total Environ. 2024, 908, 168196 10.1016/j.scitotenv.2023.168196. 37924873

[ref13] Azdarpour A.; Afkhami Karaei M.; Hamidi H.; Mohammadian E.; Honarvar B. CO2 Sequestration through Direct Aqueous Mineral Carbonation of Red Gypsum. Petroleum 2018, 4 (4), 398–407. 10.1016/j.petlm.2017.10.002.

[ref14] Lachehab A.; Mertah O.; Kherbeche A.; Hassoune H. Utilization of Phosphogypsum in CO2 mineral Sequestration by Producing Potassium Sulphate and Calcium Carbonate. Mater. Sci. Energy Technol. 2020, 3, 611–625. 10.1016/j.mset.2020.06.005.

[ref15] Wang B.; Pan Z.; Cheng H.; Zhang Z.; Cheng F. A Review of Carbon Dioxide Sequestration by Mineral Carbonation of Industrial Byproduct Gypsum. J. Clean. Prod. 2021, 302, 126930 10.1016/j.jclepro.2021.126930.

[ref16] Pan S.-Y.; Chen Y.-H.; Fan L.-S.; Kim H.; Gao X.; Ling T.-C.; Chiang P.-C.; Pei S.-L.; Gu G. CO2 mineralization and Utilization by Alkaline Solid Wastes for Potential Carbon Reduction. Nat. Sustain. 2020, 3 (5), 399–405. 10.1038/s41893-020-0486-9.

[ref17] Gadikota G. Carbon Mineralization Pathways for Carbon Capture, Storage and Utilization. Commun. Chem. 2021, 4 (1), 1–5. 10.1038/s42004-021-00461-x. 36697549 PMC9814416

[ref18] Jun Y.-S.; Giammar D. E.; Werth C. J. Impacts of Geochemical Reactions on Geologic Carbon Sequestration. Environ. Sci. Technol. 2013, 47 (1), 3–8. 10.1021/es3027133. 23130971

[ref19] Roy S.; Edwards M. A. Preventing Another Lead (Pb) in Drinking Water Crisis: Lessons from the Washington D.C. and Flint MI Contamination Events. Curr. Opin. Environ. Sci. Health 2019, 7, 34–44. 10.1016/j.coesh.2018.10.002.

[ref20] Triantafyllidou S.; Edwards M. Lead (Pb) in Tap Water and in Blood: Implications for Lead Exposure in the United States. Crit. Rev. Environ. Sci. Technol. 2012, 42 (13), 1297–1352. 10.1080/10643389.2011.556556.

[ref21] Cánovas C. R.; Quispe D.; Macías F.; Callejón-Leblic B.; Arias-Borrego A.; García-Barrera T.; Nieto J. M. Potential Release and Bioaccessibility of Metal/Loids from Mine Wastes Deposited in Historical Abandoned Sulfide Mines. Environ. Pollut. 2023, 316, 120629 10.1016/j.envpol.2022.120629. 36370976

[ref22] Miler M.; Bavec Š.; Gosar M. The Environmental Impact of Historical Pb-Zn Mining Waste Deposits in Slovenia. J. Environ. Manage. 2022, 308, 114580 10.1016/j.jenvman.2022.114580. 35124317

[ref23] Guo Z.; Yang J.; Li K.; Shi J.; Peng Y.; Sarkodie E. K.; Miao B.; Liu H.; Liu X.; Jiang L. Leaching Behavior of As and Pb in Lead–Zinc Mining Waste Rock under Mine Drainage and Rainwater. Toxics 2023, 11 (11), 943 10.3390/toxics11110943. 37999595 PMC10675770

[ref24] Kim J. J.; Lee S. S.; Fenter P.; Myneni S. C. B.; Nikitin V.; Peters C. A. Carbonate Coprecipitation for Cd and Zn Treatment and Evaluation of Heavy Metal Stability Under Acidic Conditions. Environ. Sci. Technol. 2023, 57 (8), 3104–3113. 10.1021/acs.est.2c07678. 36781166 PMC9979612

[ref25] Hunter H. A.; Ling F. T.; Peters C. A. Coprecipitation of Heavy Metals in Calcium Carbonate from Coal Fly Ash Leachate. ACS EST Water 2021, 1 (2), 339–345. 10.1021/acsestwater.0c00109.

[ref26] Tang Y.; Elzinga E. J.; Jae Lee Y.; Reeder R. J. Coprecipitation of Chromate with Calcite: Batch Experiments and X-Ray Absorption Spectroscopy. Geochim. Cosmochim. Acta 2007, 71 (6), 1480–1493. 10.1016/j.gca.2006.12.010.

[ref27] Astilleros J. M.; Godelitsas A.; Rodríguez-Blanco J. D.; Fernández-Díaz L.; Prieto M.; Lagoyannis A.; Harissopulos S. Interaction of Gypsum with Lead in Aqueous Solutions. Appl. Geochem. 2010, 25 (7), 1008–1016. 10.1016/j.apgeochem.2010.04.007.

[ref28] Li X.; Azimzadeh B.; Martinez C. E.; McBride M. B. Pb Mineral Precipitation in Solutions of Sulfate, Carbonate and Phosphate: Measured and Modeled Pb Solubility and Pb2+ Activity. Minerals 2021, 11 ( (6), ). 620 10.3390/min11060620.

[ref29] Marani D.; Macchi G.; Pagano M. Lead Precipitation in the Presence of Sulphate and Carbonate: Testing of Thermodynamic Predictions. Water Res. 1995, 29 (4), 1085–1092. 10.1016/0043-1354(94)00232-V.

[ref30] Kim Y.; Abdilla B.; Yuan K.; De Andrade V.; Sturchio N. C.; Lee S. S.; Fenter P. Replacement of Calcium Carbonate Polymorphs by Cerussite. ACS Earth Space Chem. 2021, 5 (9), 2433–2441. 10.1021/acsearthspacechem.1c00177.

[ref31] Yuan K.; Lee S. S.; De Andrade V.; Sturchio N. C.; Fenter P. Replacement of Calcite (CaCO _3_) by Cerussite (PbCO_3_). Environ. Sci. Technol. 2016, 50 (23), 12984–12991. 10.1021/acs.est.6b03911. 27767299

[ref32] Zhu Y.; Nong P.; Zhu Z.; Pan S.; Liu H.; Deng H.; Tang S.; Zhang L. Dissolution and Solubility of Pb-Substituted Calcite, Ca-Substituted Cerussite and Their Mixtures at 25 °C. Chem. Geol. 2023, 635, 121614 10.1016/j.chemgeo.2023.121614.

[ref33] Lee H. Y. Preparation of Basic Lead Carbonate from Lead Dust by Hydrometallurgical Processes. Hydrometallurgy 2009, 96 (1–2), 103–107. 10.1016/j.hydromet.2008.08.013.

[ref34] Prieto M.; Astilleros J. M.; Fernandez-Diaz L. Environmental Remediation by Crystallization of Solid Solutions. Elements 2013, 9 (3), 195–201. 10.2113/gselements.9.3.195.

[ref35] Rosenberg Y. O.; Sadeh Y.; Metz V.; Pina C. M.; Ganor J. Nucleation and Growth Kinetics of RaxBa1–xSO4 Solid Solution in NaCl Aqueous Solutions. Geochim. Cosmochim. Acta 2014, 125, 290–307. 10.1016/j.gca.2013.09.041.

[ref36] Glynn P. Solid-Solution Solubilities and Thermodynamics: Sulfates, Carbonates and Halides. Rev. Mineral. Geochem. 2000, 40 (1), 481–511. 10.2138/rmg.2000.40.10.

[ref37] Railsback B. Stability and Solubility of Carbonate Minerals of Divalent Cations. In Some Fundamentals of Mineralogy and Geochemistry, 2020

[ref38] Abdilla B.; Lee S. S.; Fenter P.; Sturchio N. C. Dynamic Surface Incorporation of Pb2+ Ions at the Actively Dissolving Calcite (104) Surface. Environ. Sci. Technol. 2024, 58 (37), 16525–16534. 10.1021/acs.est.4c03567. 39235261

[ref39] Callagon E.; Fenter P.; Nagy K. L.; Sturchio N. C. Incorporation of Pb at the Calcite (104)–Water Interface. Environ. Sci. Technol. 2014, 48 (16), 9263–9269. 10.1021/es5014888. 25007415

[ref40] Bovenkamp G. L.; Prange A.; Schumacher W.; Ham K.; Smith A. P.; Hormes J. Lead Uptake in Diverse Plant Families: A Study Applying X-Ray Absorption Near Edge Spectroscopy. Environ. Sci. Technol. 2013, 47 (9), 4375–4382. 10.1021/es302408m. 23517351

[ref41] Barrett J. E. S.; Taylor K. G.; Hudson-Edwards K. A.; Charnock J. M. Solid-Phase Speciation of Pb in Urban Road Dust Sediment: A XANES and EXAFS Study. Environ. Sci. Technol. 2010, 44 (8), 2940–2946. 10.1021/es903737k. 20337471

[ref42] Funasaka K.; Tojo T.; Katahira K.; Shinya M.; Miyazaki T.; Kamiura T.; Yamamoto O.; Moriwaki H.; Tanida H.; Takaoka M. Detection of Pb-LIII Edge XANES Spectra of Urban Atmospheric Particles Combined with Simple Acid Extraction. Sci. Total Environ. 2008, 403 (1–3), 230–234. 10.1016/j.scitotenv.2008.05.020. 18593638

[ref43] Yu Y. H.; Tyliszczak T.; Hitchcock A. P. Pb L3 EXAFS and Near-Edge Studies of Lead Metal and Lead Oxides. J. Phys. Chem. Solids 1990, 51 (5), 445–451. 10.1016/0022-3697(90)90182-F.

[ref44] Bovenkamp G. L.; Prange A.; Roy A.; Schumacher W.; Hormes J. X-Ray Absorption Near Edge Structure Spectra as a Basis for the Speciation of Lead. J. Phys. Conf. Ser. 2009, 190 (1), 012190 10.1088/1742-6596/190/1/012190.

[ref45] Vorwerk C.; Hartmann C.; Cocchi C.; Sadoughi G.; Habisreutinger S. N.; Félix R.; Wilks R. G.; Snaith H. J.; Bär M.; Draxl C. Exciton-Dominated Core-Level Absorption Spectra of Hybrid Organic–Inorganic Lead Halide Perovskites. J. Phys. Chem. Lett. 2018, 9 (8), 1852–1858. 10.1021/acs.jpclett.8b00378. 29569928

[ref46] Virga S.; Longo A.; Pipitone C.; Giannici F. Structural Features Governing the Near-Edge X-Ray Absorption Spectra of Lead Halide Perovskites. J. Phys. Chem. C 2023, 127 (36), 18058–18066. 10.1021/acs.jpcc.3c03604.

[ref47] Parkhurst D. L.; Appelo C. A. J. Description of Input and Examples for PHREEQC Version 3: A Computer Program for Speciation, Batch-Reaction, One-Dimensional Transport, and Inverse Geochemical Calculations; Techniques and Methods; Report 6-A43: Reston, VA, 2013; p 519. 10.3133/tm6A43.

[ref48] Eary L. E.; Jenne E. A. Version 4. 00 of the MINTEQ Geochemical Code; Pacific Northwest Laboratory: Richland, WA, 1992. 10.2172/7073252.

[ref49] Yu L.; Daniels L. M.; Mulders J. J. P. A.; Saldi G. D.; Harrison A. L.; Liu L.; Oelkers E. H. An Experimental Study of Gypsum Dissolution Coupled to CaCO3 Precipitation and Its Application to Carbon Storage. Chem. Geol. 2019, 525, 447–461. 10.1016/j.chemgeo.2019.08.005.

[ref50] Li L.; Yan H.; Xu W.; Yu D.; Heroux A.; Lee W.-K.; Campbell S. I.; Chu Y. S. PyXRF: Python-Based X-Ray Fluorescence Analysis Package. In X-Ray Nanoimaging: Instruments and Methods III; SPIE, 2017; Vol. 10389, pp 38–45. 10.1117/12.2272585.

[ref100] Schindelin J.; Arganda-Carreras I.; Frise E.; Kaynig V.; Longair M.; Pietzsch T.; Preibisch S.; Rueden C.; Saalfeld S.; Schmid B.; Tinevez J.-Y.; White D. J.; Hartenstein V.; Eliceiri K.; Tomancak P.; Cardona A. Fiji: An Open-Source Platform for Biological-Image Analysis. Nat. Methods 2012, 9 (7), 676–682. 22743772 10.1038/nmeth.2019 PMC3855844

[ref51] Pattammattel A.; Tappero R.; Gavrilov D.; Zhang H.; Aronstein P.; Forman H. J.; O’Day P. A.; Yan H.; Chu Y. S. Multimodal X-Ray Nano-Spectromicroscopy Analysis of Chemically Heterogeneous Systems. Metallomics 2022, 14 (10), mfac078 10.1093/mtomcs/mfac078. 36208212 PMC9584160

[ref52] Ravel B.; Newville M. ATHENA, ARTEMIS, HEPHAESTUS: Data Analysis for X-Ray Absorption Spectroscopy Using IFEFFIT. J. Synchrotron Radiat. 2005, 12 (4), 537–541. 10.1107/S0909049505012719. 15968136

[ref53] US EPA. Secondary Drinking Water Standards: Guidance for Nuisance Chemicals. https://www.epa.gov/sdwa/secondary-drinking-water-standards-guidance-nuisance-chemicals (accessed 2024-11-16).

[ref54] US EPA. National Primary Drinking Water Regulations. https://www.epa.gov/ground-water-and-drinking-water/national-primary-drinking-water-regulations (accessed 2024-11-16).

[ref55] Xu C.; Nong P.; Zhu Z.; Kong Q.; Zhou X.; Deng H.; Tang S.; Zhang L.; Zhu Y. Dissolution and Solubility of the Solid Solution between Calcite and Smithsonite [(Ca1–xZnx)CO3] at 25 °C. ACS Earth Space Chem. 2023, 7 (7), 1401–1415. 10.1021/acsearthspacechem.3c00068.

[ref56] Glynn P. D.; Reardon E. J.; Plummer L. N.; Busenberg E. Reaction Paths and Equilibrium End-Points in Solid-Solution Aqueous-Solution Systems. Geochim. Cosmochim. Acta 1990, 54 (2), 267–282. 10.1016/0016-7037(90)90317-E.

[ref57] Crocket J. H.; Winchester J. W. Coprecipitation of Zinc with Calcium Carbonate. Geochim. Cosmochim. Acta 1966, 30 (10), 1093–1109. 10.1016/0016-7037(66)90119-0.

[ref58] Follner S.; Wolter A.; Helming K.; Silber C.; Bartels H.; Follner H. On the Real Structure of Gypsum Crystals. Cryst. Res. Technol. 2002, 37 (2–3), 207–218. 10.1002/1521-4079(200202)37:2/3<207::AID-CRAT207>3.0.CO;2-L.

[ref59] Beaugnon F.; Preturlan J. G. D.; Fusseis F.; Gouillart E.; Quiligotti S.; Wallez G. From Atom Level to Macroscopic Scale: Structural Mechanism of Gypsum Dehydration. Solid State Sci. 2022, 126, 106845 10.1016/j.solidstatesciences.2022.106845.

[ref60] Pedersen B. F.; Semmingsen D. Neutron Diffraction Refinement of the Structure of Gypsum, CaSO4.2H20. Acta Crystall, Sect. B 1982, 38, 1074 10.1107/S0567740882004993.

[ref61] Ulian G.; Moro D.; Valdrè G. Elastic Properties of Heterodesmic Composite Structures: The Case of Calcite CaCO3 (Space Group R 3 - c). Compos. Part C Open Access 2021, 6, 100184 10.1016/j.jcomc.2021.100184.

[ref62] Kim Y.; Lee S. S.; Abdilla B.; Nikitin V.; Sturchio N. C.; Fenter P. Formation of Zinc Carbonate Phases on Dissolving Calcite, Aragonite, and Vaterite in Acidic Aqueous Solutions. Geochim. Cosmochim. Acta 2024, 380, 131–139. 10.1016/j.gca.2024.06.034.

[ref63] Coetzee P. P.; Yacoby M.; Howell S.; Mubenga S. B. Scale Reduction and Scale Modification Effects Induced by Zn and Other Metal Species in Physical Water Treatment. Water SA 1998, 24, 77–84.

[ref64] Xu B.; Qin J.; Yi Y. Use of Ladle Slag for CO2 Sequestration and Zinc Immobilization. Resour. Conserv. Recycl. 2023, 199, 107220 10.1016/j.resconrec.2023.107220.

[ref65] Temmam M.; Paquette J.; Vali H. Mn and Zn Incorporation into Calcite as a Function of Chloride Aqueous Concentration. Geochim. Cosmochim. Acta 2000, 64 (14), 2417–2430. 10.1016/S0016-7037(00)00375-6.

[ref66] Coetzee P. P.; Yacoby M.; Howell S. The Role of Zinc in Magnetic and Other Physical Water Treatment Methods for the Prevention of Scale. Water SA 1996, 22 (4), 319–326. 10.10520/AJA03784738_1096.

[ref67] Yang F.; Zhou H.; Hu J.; Ji S.; Lai C.; Wang H.; Sun J.; Lei L. Thorn-like and Dendrite Lead Sulfate as Negative Electrode Materials for Enhancing the Cycle Performance of Lead-Acid Batteries. J. Energy Storage 2022, 49, 104112 10.1016/j.est.2022.104112.

[ref68] Shannon R. D. Revised Effective Ionic Radii and Systematic Studies of Interatomic Distances in Halides and Chalcogenides. Acta Crystallogr., Sect. A 1976, 32 (5), 751–767. 10.1107/S0567739476001551.

[ref69] Thonemann N.; Zacharopoulos L.; Fromme F.; Nühlen J. Environmental Impacts of Carbon Capture and Utilization by Mineral Carbonation: A Systematic Literature Review and Meta Life Cycle Assessment. J. Clean. Prod. 2022, 332, 130067 10.1016/j.jclepro.2021.130067.

[ref70] Di Maria A.; Snellings R.; Alaerts L.; Quaghebeur M.; Van Acker K. Environmental Assessment of CO2 mineralisation for Sustainable Construction Materials. Int. J. Greenh. Gas Control 2020, 93, 102882 10.1016/j.ijggc.2019.102882.

[ref71] Lin X.; Zhang Y.; Liu H.; Boczkaj G.; Cao Y.; Wang C. Carbon Dioxide Sequestration by Industrial Wastes through Mineral Carbonation: Current Status and Perspectives. J. Clean. Prod. 2024, 434, 140258 10.1016/j.jclepro.2023.140258.

[ref72] Zhao H.; Li H.; Bao W.; Wang C.; Li S.; Lin W. Experimental Study of Enhanced Phosphogypsum Carbonation with Ammonia under Increased CO2 Pressure. J. CO2 Util. 2015, 11, 10–19. 10.1016/j.jcou.2014.11.004.

