# Supplementary material for: Carbon Mineralization of Sulfate Wastes Containing Pb: Synchrotron Pb M3-Edge XANES Analysis of Simultaneous Heavy Metal and Carbon Sequestration
Source: Environ Sci Technol. 2025 Apr 3;59(14):7366–76. doi: 10.1021/acs.est.5c01640 (PMC12004928; doi:10.1021/acs.est.5c01640)
Supplement: Supplementary file 1 — es5c01640_si_001.pdf [file es5c01640_si_001.pdf]

Supporting Information for:

Carbon mineralization of sulfate wastes containing Pb: Synchrotron Pb M3-edge XANES analysis of simultaneous heavy metal and carbon sequestration

Jun Hu[1], Lauren N Pincus[1], Dominik Wierzbicki[2], Yonghua Du[2], Catherine A Peters[1*]

[1]Department of Civil and Environmental Engineering, Princeton University, Princeton, New Jersey, 08544, United States

[2] National Synchrotron Light Source II, Brookhaven National Lab, Upton, New York 11973, United States

*Corresponding author

This document contains 6 pages with 8 figures (Figure S1-S8) and 1 table (Table S1).

Table S1. X-ray Absorption Energy of calcium (Ca), sulfur (S), lead (Pb) and zinc (Zn)

**Element-Edge**	**Energy (KeV)**
Ca-K	4.0381
S-K	2.4720
Pb-K	88.0045
Pb-L1	15.8608
Pb-L2	15.2000
Pb-L3	13.0352
Pb-M3	3.0664
Pb-M4	2.5856
Pb-M5	2.4840
Zn-K	9.6590

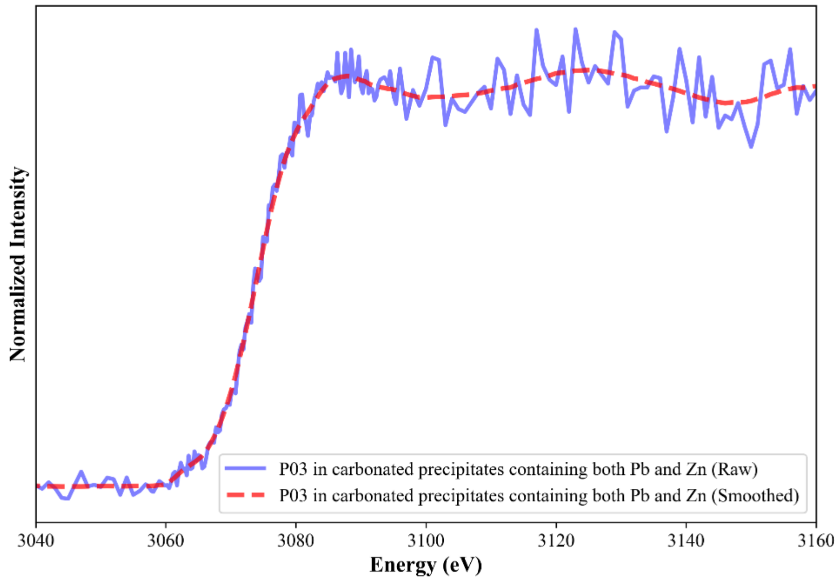

Figure S1. Pb M3-edge XANES spectra of P03 in carbonated precipitates containing both Pb and Zn. A Gaussian filter algorithm (Kernal size= 19, Width= 5) was applied to these scans to smooth data and reduce noise.

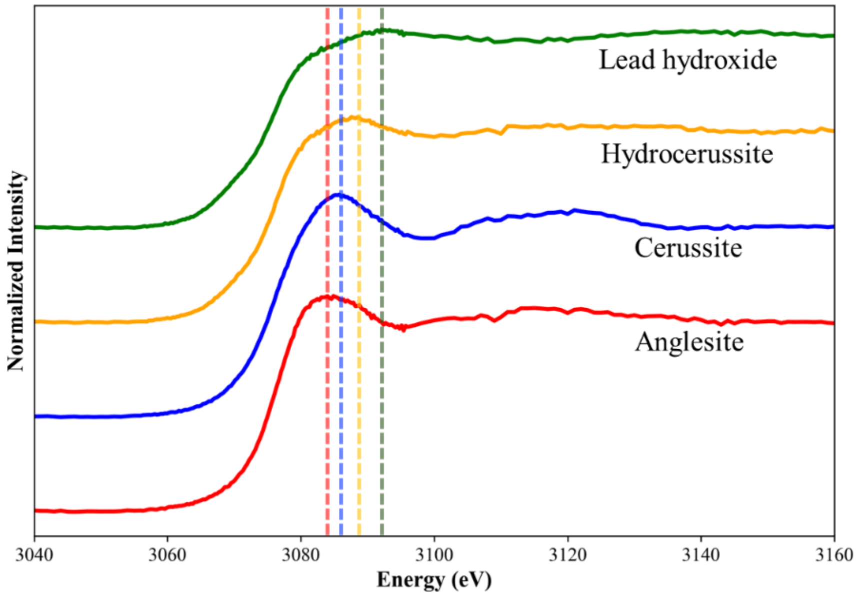

Figure S2. Pb M3-edge XANES spectra of lead reference materials: anglesite [$PbSO_4$, red], cerussite [$PbCO_3$, blue], hydrocerussite [$Pb_3(CO_3)_2(OH)_2$, yellow], and lead hydroxide [$Pb(OH)_2$, green]. All scans were acquired at the NSLS-II TES beamline, operating in the tender X-ray range (2–5.5 keV). The dashed lines indicate the absorption edge positions for each reference material: red for anglesite, blue for cerussite, yellow for hydrocerussite, and green for lead hydroxide.

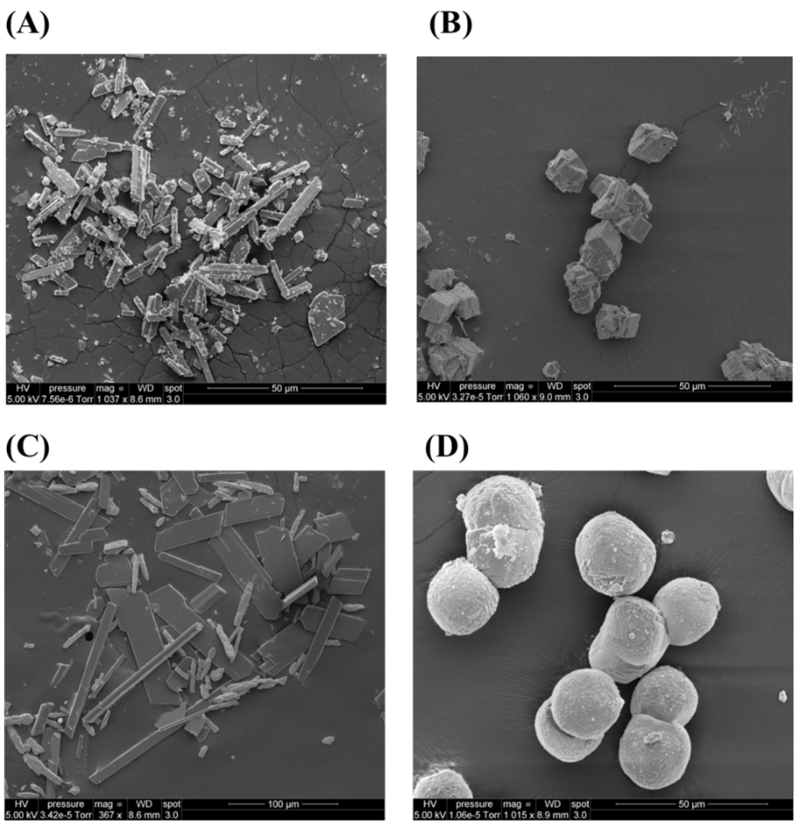

Figure S3. SEM secondary electron images of initial gypsum precipitates containing (A) only Pb and (C) both Pb and Zn; carbonated precipitates containing (B) only Pb and (D) both Pb and Zn.

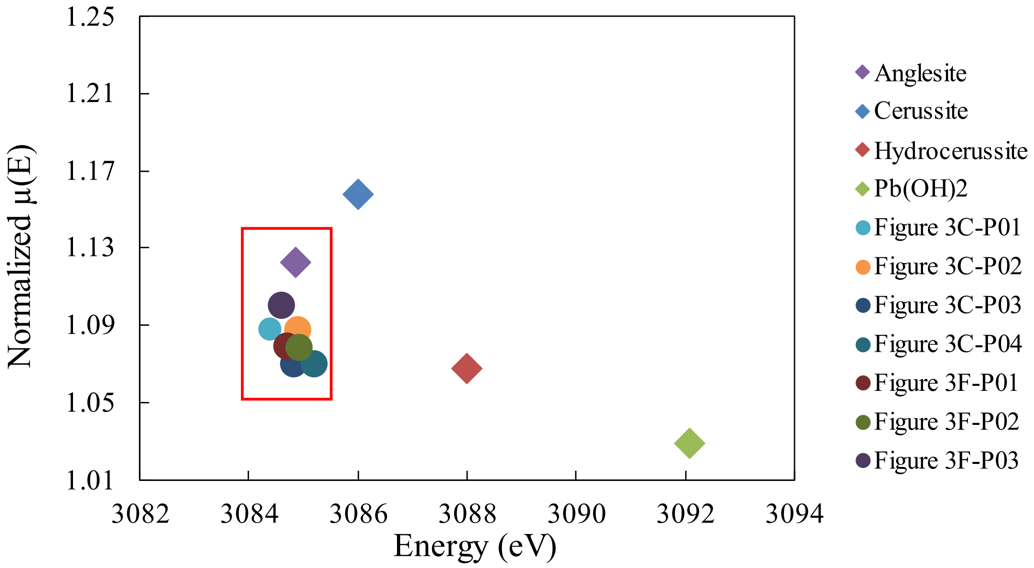

Figure S4. Absorption-edge positions (energy vs. normalized μ(E)) of selected points from Figures 3C and 3F, compared with reference standards. Rhombus symbols represent reference standards (anglesite, cerussite, hydrocerussite and $Pb(OH)_2$), while circle symbols correspond to measured points from the gypsum precipitates.

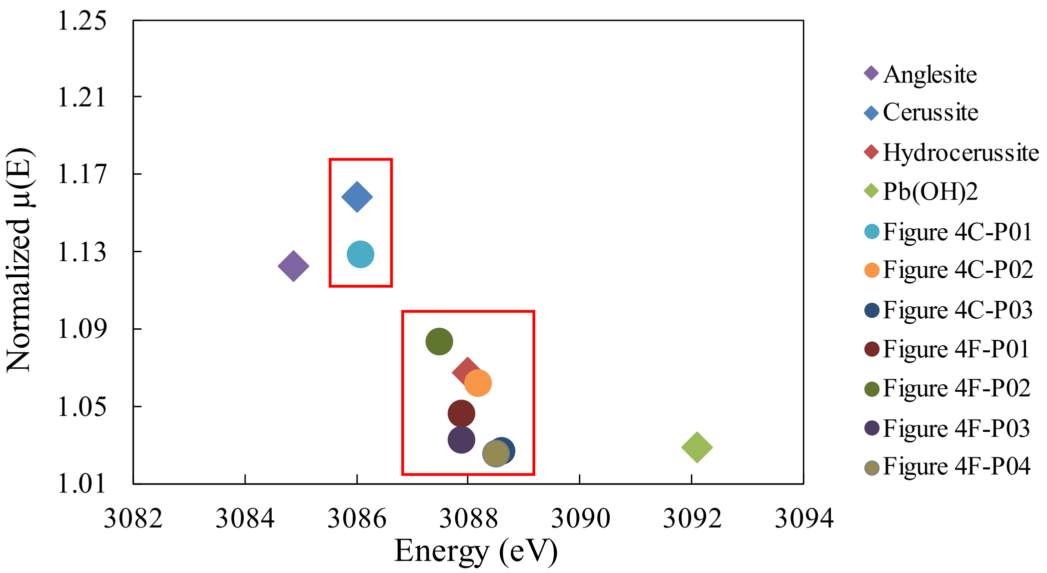

Figure S5. Absorption-edge positions (energy vs. normalized µ(E)) of selected points from Figures 4C and 4F, compared with reference standards. Rhombus symbols represent reference standards (anglesite, cerussite, hydrocerussite and $Pb(OH)_2$), while circle symbols correspond to measured points from the carbonated precipitates.

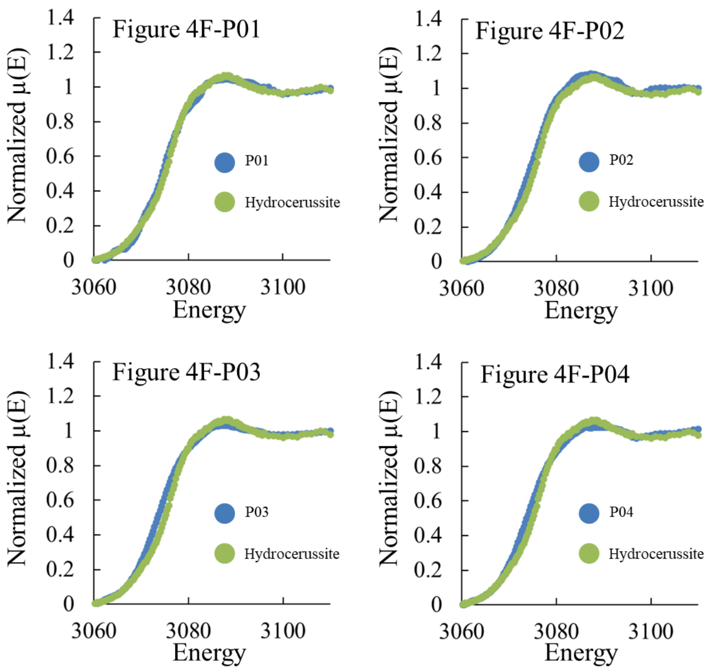

Figure S6. Overlay comparison of XANES spectra for selected points (P01–P04) in Figure 4F with the hydrocerussite reference spectrum

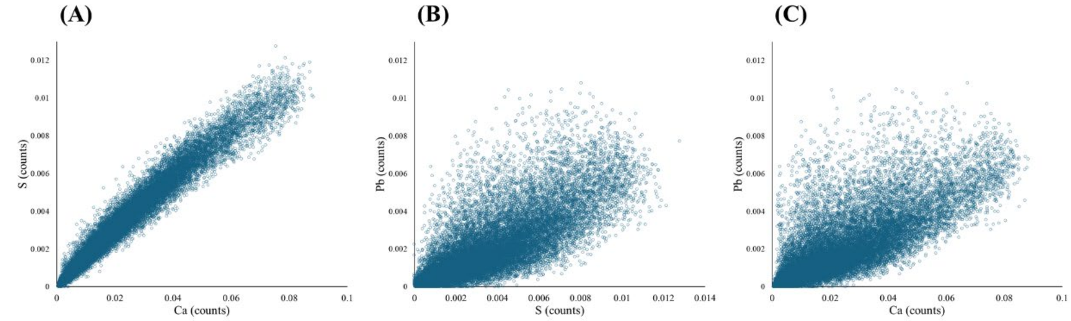

Figure S7. Element correlation plots of normalized μ-XRF counts for (A) calcium (Ca) vs. sulfur (S); (B) sulfur (S) vs. lead (Pb); and (C) calcium (Ca) vs. lead (Pb) in initial sulfate precipitates containing only Pb.

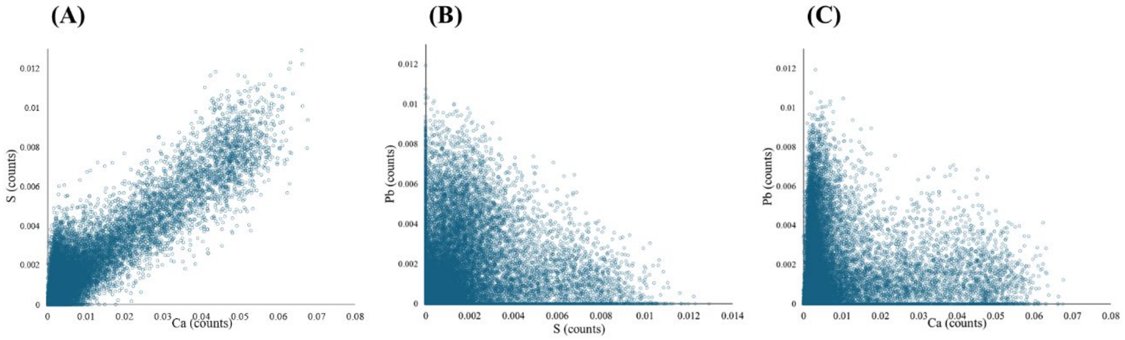

Figure S8. Element correlation plots of normalized μ-XRF counts for (A) calcium (Ca) vs. sulfur (S); (B) sulfur (S) vs. lead (Pb); and (C) calcium (Ca) vs. lead (Pb) in initial sulfate precipitates containing both Pb and Zn.
